# Clinical validation of a proposed diagnostic classification for pulpitis

**DOI:** 10.1111/iej.14254

**Published:** 2025-05-27

**Authors:** R. N. Karrar, S. G. Craig, H. F. Duncan, S. A. Abushouk, S. Y. Elfiel, F. T. Lundy, M. Clarke, I. A. El‐Karim, Reliability Assessment Group

**Affiliations:** ^1^ Faculty of Dentistry University of Khartoum Khartoum Sudan; ^2^ School of Medicine Dentistry and Biomedical Sciences Queen's University Belfast Belfast UK; ^3^ Division of Restorative Dentistry & Periodontology Dublin Dental University Hospital, Trinity College Dublin, University of Dublin Dublin Ireland

**Keywords:** cluster analysis, diagnosis, pulpitis, vital pulp treatment

## Abstract

**Aim:**

Determine the reliability and clinical validity of the Wolters classification of pulpitis.

**Methodology:**

Prospective diagnostic accuracy study in which patients with pulpitis were included. Based on history, clinical and radiographic examination, participants were categorized into initial, mild, moderate or severe pulpitis based on Wolters classification and received treatment as suggested in the classification. The American Association of Endodontists (AAE) classification was used for comparison. Treatment outcome was evaluated at 12 months. Classification reliability was assessed by measuring the interrater agreement using Fleiss' Kappa. Construct validity was assessed by cluster analysis using an unsupervised machine learning approach. Predictive validity was determined by the association of treatment outcome with the diagnostic category. All statistical analyses were conducted using R v4.3.1; a *p*‐value of <.05 was considered statistically significant.

**Results:**

Ninety‐two patients were included in the study. The interrater reliability showed fair agreement for Wolters classification (Kappa (*κ*) = 0.593, 95% confidence interval (CI): 0.592–0.595), compared with substantial agreement (*κ* = 0.888, 95% CI: 0.887–0.889) for the AAE classification. Association of Wolters classification with output of unsupervised K‐mode cluster analysis showed that the use of a three‐category model may improve discrimination of pulpitis subdivided into mild, moderate, and severe categories. The revised classification model demonstrated 100% sensitivity and specificity for accurate discrimination between mild and severe pulpitis and 83% sensitivity and 85.7% specificity for classifying moderate pulpitis. There was no significant difference between these revised diagnostic categories considering treatment outcome, suggesting good predictive validity (*p* > .05). Compared with the AAE classification, the new classification in conjunction with proposed treatments resulted in preserving 87% of the pulps compared with historical treatment.

**Conclusions:**

We propose that a revised Wolters classification model is suitable for determining mild, moderate, and severe pulpitis to aid in clinical management of pulpitis.

## INTRODUCTION

Pulpitis is an inflammatory disease of the dental pulp, predominantly caused by oral bacteria. It involves complex signalling cascades that reflect the acute and chronic immunological inflammatory response (Cooper et al., 2017; El Karim et al., 2021). However, obtaining a clinical diagnosis of pulpitis that accurately reflects the pathological status of the dental pulp has proven problematic for many reasons. These include the accessibility of the dental pulp tissue, unpredictability of subjective measures, and limitations of current diagnostic methods (Donnermeyer et al., 2023; Mejàre et al., 2012).

Although histological examination is considered the gold standard diagnostic test for pulpitis, attempts to correlate inflammatory changes histologically with clinical findings have generally demonstrated a lack of consistent agreement (Baume, 1970; Dummer et al., 1980), with only one study highlighting good correlation (Ricucci et al., 2014). Furthermore, if pulp preservation is indicated, histological examination is not clinically feasible or appropriate. Therefore, classifications based on clinical signs, symptoms and sensibility tests are more pragmatic and clinically useable (Abbott & Yu, 2007). The widely used American Association of Endodontists (AAE) diagnostic classification, which is based on signs and symptoms (AAE, 2009), describes pulpitis as reversible, indicating that the inflammation should resolve following appropriate management or irreversible, where the inflamed pulp is considered incapable of healing and root canal treatment is indicated. Several reports have demonstrated high success of pulpotomy for teeth with a clinical diagnosis of symptomatic irreversible pulpitis (Cushley et al., 2019; Elmsmari et al., 2021), which has led to vital pulp treatment (VPT) being recommended by global professional societies such as the European Society of Endodontology (ESE) and AAE (AAE, 2021; Duncan et al., 2023; ESE et al., 2019). The preservation of some pulp tissue in teeth with a clinical diagnosis indicating irreversible damage supports the notion that pulpal inflammation exists on a continuum rather than dichotomous discrete states influenced by caries depth and activity (Bjørndal et al., 2019; ESE et al., 2019). This renders the term ‘irreversible’ outdated and obsolete. As a result, editorials from leading groups have called for changes to the existing diagnostic classification for pulpal disease (Galicia & Peters, 2021; Rechenberg & Zehnder, 2020; Wolters et al., 2017).

One of these editorials (Wolters et al., 2017) proposed a new classification of pulpitis, describing the disease as ‘initial’, ‘mild’, ‘moderate’ and ‘severe’ based on specific detailed symptoms, signs and findings of clinical tests. Notably, a treatment modality was assigned to each category within their diagnostic criteria. ‘Wolters’ classification offered the opportunity to prescribe VPTs to teeth that would previously have been treated by either root canal treatment or extraction. Not uncommonly within endodontics, however, Wolters' proposal was based on expert opinions and not developed through a moderated consensus or validated process. One recent study (Careddu & Duncan, 2021) evaluated the success of partial pulpotomy after 1 year whilst comparing Wolters classification to the established AAE classification and concluded that subdividing pulpitis into moderate and severe could offer a prognostic advantage over the AAE classification. However, we are not aware of any prospective clinical studies that assessed the reliability and clinical validity of the proposed classification in clinical practice.

The development of scale‐based classifications requires validation before adoption in clinical practice (Elman et al., 2020; Ford et al., 2007). Three types of validity are generally tested in this context: content or face validity, construct validity (the degree to which a category can be discriminated from other categories based on signs and symptoms and clinical findings) and criterion or predictive validity (evaluation of the ability of a classification system to predict a future outcome). Validation and diagnostic criteria assessment requires obtaining a detailed clinical evaluation including validated pain history questionnaires (Van Wijk & Hoogstraten, 2002) and a standardized clinical examination process. Using this approach, this study aims to determine:

1. The reliability of the classification by measuring interrater agreement in making the diagnosis using patient history, clinical, and radiographic examination data.
2. The face validity of the classification is assessed by determining whether the suggested criteria for initial, mild, moderate or severe pulpitis are recorded by the patient with respect to reported symptoms and clinical examination findings.
3. The construct validity of the classification using cluster analysis.
4. The predictive validity of the classification using 12‐month treatment outcome data as proxy standard.

## METHODS

### Study design

A prospective longitudinal validation accuracy study in which patients with caries and suspected pulpal involvement were screened for inclusion under specific diagnostic criteria suggested by Wolters et al. (2017).

This observational study has been written according to Preferred Reporting items for Observational studies in Endodontics (PROBE) 2023 guidelines.

### Sample size calculation

The sample size was calculated using (Buderer, 1996) methods based on hypothesized 95% sensitivity, 85% specificity, 0.1 precision error, 95% CI, 35% disease prevalence and 15% expected dropout rate. This showed that a total of 90 participants were required.

### Inclusion criteria

Patients aged 18 years and over, presenting with deep caries (caries reaching the inner quarter of dentine but with a zone of hard or firm dentine between the caries and the pulp), or extremely deep carious lesions (caries penetrating the entire thickness of the dentine, where pulp exposure is unavoidable during operative treatment) (ESE et al., 2019). Teeth should be responsive to sensibility tests. Only permanent teeth that were restorable and could be adequately isolated with dental dam were included.

### Exclusion criteria

Teeth with clinical symptoms of pulp necrosis and signs of apical periodontitis, mobility, periodontitis (pocket depth ≥ 4 mm), crowded teeth (due to difficulties in sensibility testing) and teeth with internal/external resorption identified in radiographs were excluded. Patients unable to provide written consent were also excluded from the study.

### The diagnostic process: History and clinical examination

A detailed history of presenting complaints was performed using a structured questionnaire modified from the McGill dental pain questionnaire (Van Wijk & Hoogstraten, 2002) on pain history (onset, character, spontaneous or provoked, duration, continuity, localized or diffused, provoking factors and associated symptoms and signs). A complete clinical examination was carried out, including dental hard tissue charting and periodontal and soft tissue examination. The sensibility test was carried out with a −50°C thermal test (Endofrost, Coltene, Cuyahoga Falls, OH, USA), and intra‐oral periapical radiographs were also obtained as part of the diagnostic procedure. Clinical examination and radiographic assessment were performed by a single experienced endodontist to ensure consistency.

### Allocation of patients to respective diagnostic category and provision of treatment

Following history, clinical and radiographic examination, patients were categorized into initial, mild, moderate or severe pulpitis based on Wolters classification to receive corresponding treatment as proposed in the classification (Table 1). Prior to the study, the endodontist who applied the classification criteria underwent extensive calibration exercises to ensure the appropriate application of the criteria. Ambiguous cases were discussed with senior endodontists involved in developing the Wolters' classification criteria. Patients were also classified using the AAE classification as reference (i.e. reversible and irreversible pulpitis). All treatments described by Wolters et al. (2017) were carried out by a trained endodontist under magnification and dental dam isolation (Sanctuary Health, Perak, Malaysia). Teeth were anaesthetized with 2% lidocaine hydrochloride and 1/80 000 epinephrine. Specific treatment procedures were:

**TABLE 1:** Wolters proposed pulp classification (Wolters et al., 2017).

Diagnosis category	Symptoms/criteria	Hypothesis/construct	Suggested treatment
Initial pulpitis	Heightened (++) but not lengthened response to the cold test Not sensitive upon percussion No spontaneous pain	Not provided	Indirect pulp treatment (IPT)
Mild pulpitis	Heightened and lengthened reaction to cold (+++), warmth and sweet stimuli that can last up to 20 seconds but then subsides, possibly percussion sensitive (−/+)	It would be implied that there is limited local inflammation confined to the crown pulp	As above
Moderate pulpitis	Clear symptoms, strong, heightened and prolonged reaction to cold, which can last for minutes, possibly percussion sensitive (−/+) spontaneous, dull pain that can be suppressed with pain medication	It would be implied that there is extensive Local inflammation confined to the crown pulp	Partial or complete/full pulpotomy
Severe pulpitis	Severe spontaneous pain Clear pain reaction to warmth and cold stimuli Often, sharp to dull throbbing pain, patients have trouble sleeping because of the pain (gets worse when lying down). Tooth is very sensitive to touch and percussion	It would be implied that there is extensive local inflammation in the crown pulp that possibly extends into the root canals	Complete/full pulpotomy or pulpectomy depending on control of bleeding. No time limit was set to achieve haemostasis

*Selective caries removal*: As described by Wolters et al. (2017), the procedure was performed in teeth with deep caries and symptoms indicative of initial or mild pulpitis. This treatment aims to avoid pulp exposure. All carious dentine was removed from the enamel/dentine junction, but on the axial/pulpal wall, the bulk of soft infected dentine was removed, leaving a soft layer of affected dentine, using a single‐step selective caries removal. A hydraulic calcium silicate cement Biodentine (Septodont Ltd., Saint Maur des Fausse's, France) was placed to a thickness of at least 3 mm prior to placement of the final resin‐based composite restoration (3M™, Bracknell, UK).

*Partial pulpotomy*: As described in the classification, partial pulpitis was indicated for cases with a clinical diagnosis of moderate pulpitis. The procedure involved complete (non‐selective) caries removal and exposure of the dental pulp, followed by removal of 1–2 mm of pulp tissue at the exposure site. The pulp was then rinsed with 2% NaOCl, and bleeding was controlled by applying a cotton pellet soaked in NaOCl for up to 10 min prior to the placement of Biodentine. If bleeding was not controlled, further pulp tissue was removed until haemostasis was achieved. Biodentine was then placed to dress the pulp and fill the entire cavity. After 7 days, the tooth was restored with a resin‐based composite restoration.

*Complete/full pulpotomy*: This procedure is recommended for teeth with a clinical diagnosis of severe pulpitis and is carried out in a similar manner to partial pulpotomy, except that the entire coronal pulp is removed. Bleeding was controlled by the application of a cotton pellet soaked in 2% NaOCl for up to 10 min. A layer of Biodentine was then placed to dress the pulp and fill the entire cavity. After 7 days, the tooth was restored with a resin‐based composite restoration. For cases diagnosed with severe pulpitis but discovered to be partially necrotic, pulpectomy and subsequent root canal treatment were carried out.

### Clinical evaluation

The success of the treatment was based on composite outcomes, including both clinical and radiographic data. Clinical success criteria are defined as the absence of pain suggestive of pulpitis or apical periodontitis, no tenderness to palpation or percussion and no sign of infection (swelling or sinus tract). Pain was assessed using a numerical rating scale (NRS). A positive response to the sensibility test in cases treated with selective caries removal and partial pulpotomy was recorded, but not for those treated with full pulpotomy (ESE et al., 2019). Radiographic assessment was carried out by an independent assessor (experienced endodontist). Radiographic success criteria included the absence of periapical changes, that is, PAI score 1 or 2 (Ørstavik et al., 1986).

### Reliability assessment

To assess the reproducibility of the classification, anonymized detailed clinical data, including symptoms and findings of clinical and radiographic examination, was assessed independently by five experienced endodontists and two experienced general dental practitioners with a clinical and research interest in VPT. Each rater was asked to assign every patient into initial, mild, moderate or severe pulpitis based on the history and clinical and radiographic examination data that was tabulated in an Excel sheet for every patient. The rater was also asked to concomitantly assign patients according to the AAE classification categories for comparison. The inter‐rater reliability was then calculated for each classification for all the assessors using Fleiss' Kappa statistics (Gisev et al., 2013).

### Validity assessment

#### Face validity

This refers to how the criteria used to describe the respective diagnostic categories appeared to fit each specified category (Zanarini et al., 1991). Face validity was calculated by recording the occurrence of positive patient responses to each variable (signs or symptoms) described by Wolters et al. (2017) in each diagnostic category. For example, the frequency of stimulated pain and the presence of a heightened response to the cold test in mild pulpitis (Table 1). The proportion of patients who responded to the various items described by Wolters and the AAE classifications during clinical examination in each diagnostic category was calculated.

#### Construct (discriminative) validity

This identifies whether the theoretical diagnostic structure is supported by the data (Berntson et al., 2001; Ford et al., 2007). To identify the underlying dimensions or the construct of the categories, cluster analysis was performed on the recorded variables to confirm the number of categories. K‐mode clustering, an unsupervised machine learning method amenable to clustering categorical data, was used to determine the number of distinct clusters that best explain the symptoms recorded. The accuracy of the revised Wolters classification was then assessed against the optimum number of K‐mode clusters by calculation of sensitivity, specificity, positive predictive value (PPV) and negative predictive value (NPV).

#### Predictive validity of the classification

This was determined by assessing treatment outcomes for each diagnostic category as described above. The treatment success was defined as a composite measure of clinical and radiographic success. The association of the treatment outcome with the diagnostic category was measured statistically using the chi‐square test.

### Statistical analysis

Data were analysed using R v4.3.1 with the KlaR, ggplot2, and base packages installed. Cluster analysis was performed with the K‐modes algorithm for categorical variables. The number of clusters was determined quantitatively using the elbow method, the total within‐cluster distance and how meaningful the cluster is with respect to the variables of interest in order to maximize variability and explainability without overfitting. Chi‐square and Kruskal–Wallis Rank‐Sum tests were used to assess the association between categorical and continuous variables in each cluster. Fleiss' Kappa (*κ*) was measured to assess inter‐rater reproducibility, with values <0.00 = poor; 0–0.2 = slight; 0.21–0.4 = fair; 0.41–0.6 = moderate; 0.61–0.8 = substantial; and 0.81–1 = almost perfect. A *p*‐value of <.05 was considered statistically significant.

## RESULTS

A total of 92 patients who satisfied the inclusion criteria were included in this study. The average age was 27.9 years (SD: ±8.3). There were 71 female and 21 male participants. Only posterior teeth were included (69 molars and 23 premolars) in the final sample. Based on the signs and symptoms as described by Wolters et al. (2017) (Table 1), patients were categorized into initial (*n* = 17) mild (*n* = 15), moderate (*n* = 30) and severe pulpitis (n = 30). As per the AAE classification, patients were categorized as reversible pulpitis (*n* = 32) and irreversible pulpitis (*n* = 60).

### Reliability assessment

To assess the degree of reproducibility when the classification criteria were used in routine practice, five endodontists and two experienced dentists independently assigned patients to each diagnostic category for both the Wolters classification (Wolters et al., 2017) and the AAE classification (AAE, 2009) classifications based on the symptoms and clinical data, as described above. The interrater reliability was assessed by calculation of Fleiss' Kappa. The overall agreement for the different categories of Wolter's classification was fair (*κ* = 0.593, 95% CI: 0.592–0.595, *p* = .0001). The agreement was moderate for initial pulpitis (*κ* = 0.553, 95% CI: 0.551–0.555, *p* = .0001) and severe pulpitis (*κ* = 0.585, 95% CI: 0.583–0.587, *p* = .0001), substantial for mild (*κ* = 0.736, 95% CI: 0.734–0.738, *p* = .0001) and fair for moderate pulpitis (*κ* = 0.494, 95% CI: 0.493–0.496, *p* = .0001). When the clinicians classified the patients using the AAE classification, the overall agreement was substantial (*κ* = 0.888, 95% CI: 0.887–0.889, *p* = .0001) with reversible pulpitis reaching near perfect agreement (*κ* = 0.85, 95% CI: 0.857–0.859, *p* = .0001) and irreversible pulpitis reaching substantial agreement (*κ* = 0.75, 95% CI: 0.749–0.751, *p* = .0001).

### Face validity

The classification proposed specific signs and symptoms to define initial, mild, moderate and severe categories. The face validity for these categories was assessed by counting the frequency of each patient's reported signs and symptoms, as shown in Table 2a–d. In the ‘initial pulpitis’ group (Table 2a), stimulated pain was reported by 82% (14/17), none reported spontaneous pain and 18% (3/17) reported no history of pain at all. All cases had deep caries and showed heightened but not prolonged response to cold. In the ‘mild pulpitis’ group (Table 2b), stimulated pain was reported by 86% (13/15) and no pain by 14% (2/15). In This group 80% (12/15) cases showed heightened, but not prolonged (less than 20 s) reaction to cold, whilst 20% (3/15), showed strong clear heightened response that last for less than 20 s. All cases had deep caries lesions except three that had extremely deep caries. The overall face validity for these categories is good, except that several patients were asymptomatic, which is not described in the classification.

**TABLE 2:** Face/content validity of the classification: The report and frequency of each item (symptom/sign or test results) are tabulated for each category.

Criterion	Frequency
**(a) Face validity: Initial pulpitis**	
*Symptoms*	
Pain stimulated	14/17 (82%)
Pain spontaneous	0/17 (0%)
Asymptomatic	3/17 (18%)
*Examination*	
Heightened NOT lengthened reaction to cold	17/17 (100%)
Strong clear Heightened NOT lengthened reaction to cold	0/17 (0%)
Percussion sensitive (slight)	0/17 (0%)
Deep caries	17/17 (100%)
Extremely deep caries	0/17 (0%)
**(b) Face validity: Mild pulpitis**	
*Symptoms*	
Pain stimulated	13/15 (86%)
Pain spontaneous	0/15 (0%)
Asymptomatic	2/15 (14%)
*Examination*	
Heightened NOT lengthened reaction to cold	12/15 (80%)
Strong clear Heightened NOT lengthened reaction to cold	3/15 (20%)
Percussion sensitive (slight)	3/15 (20%)
Deep caries	12/15 (80%)
Extremely deep caries	3/15 (20%)
**(c) Face validity: Moderate pulpitis**	
*Symptoms*	
Pain stimulated	18/30 (60%)
Pain spontaneous	11/30 (37%)
Asymptomatic	1/30 (3%)
*Examination*	
Strong, clear heightened and prolonged reaction to cold, which can last for minutes	14/30 (47%)
Heightened and prolonged reaction to cold, which can last for minutes	16/30 (53%)
Percussion sensitive	7/30 (23%)
Deep caries	15/30 (50%)
Extremely deep caries	15/30 (50%)
**(d) Face validity: Severe pulpitis**	
*Symptoms*	
Pain stimulated	0/30 (0%)
Severe spontaneous pain and clear pain reaction to warmth and cold stimuli	30/30 (100%)
Sleep disturbing /lay down	30/30 (100%)
Asymptomatic	0/30 (0%)
*Examination*	
Heightened and prolonged reaction to cold, which can last for minutes	4/30 (13%)
Strong, clear, heightened and prolonged reaction to cold, which can last for minutes	23/30 (77%)
Delayed heightened response, which can last for minutes	3/30 (10%)
Percussion sensitive	16/30 (53%)
Deep caries	4/30 (13%)
Extremely deep caries	26/30 (87%)

The ‘moderate pulpitis’ group (Table 2c) included 60% (18/30) with stimulated pain, 37% (11/30) with spontaneous pain and 3% (1/30) with no pain. Just over half (16/30) showed a heightened and prolonged response to the cold test (minutes), whilst 47% (14/30) showed a strong, clear, heightened and prolonged response to the cold test. Sensitivity to percussion was reported in 23% (7/30). The overall face validity of this category is compromised by the reported lack of spontaneous pain in 37% (11/30) and no history of pain in 3% (1/30) of participants. Caries depth assessment, which was also not included in the classification, was classified as deep in 50% (15/30) and extremely deep in the other 50% (15/30).

The ‘severe pulpitis’ category is defined by the presence of severe spontaneous pain, clear pain reaction to warmth and cold stimuli, often sharp to dull throbbing pain, trouble sleeping and teeth that are very sensitive to touch and percussion. As shown in Table 2c, 100% (30/30) of patients presented with symptoms of severe spontaneous pain and all had sleep disturbance. Sensitivity to percussion was observed in 53% (26/30). Radiographically, 87% (26/30) had extremely deep caries and 13% (4/30) had deep caries. In 10% (3/30), the response to cold was delayed, but the reaction was prolonged to minutes with severe lingering pain. These cases were discovered to have partially necrotic pulps after access to the pulp chamber and received pulpectomy and root canal treatment instead of complete pulpotomy. The overall face validity of this category is good, but the descriptors are compromised by the delayed response to cold and percussion sensitivity in only 53% (16/30) of cases.

### Construct (discriminative) validity

**FIGURE 1:**
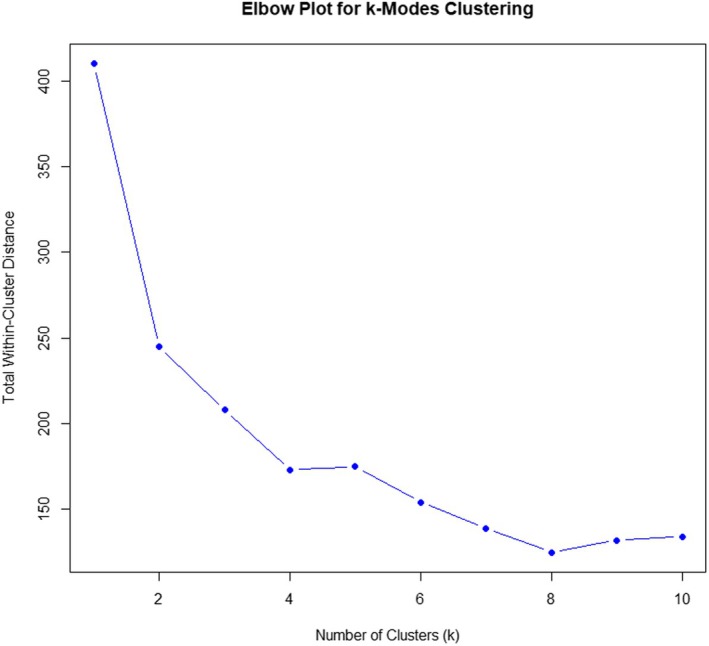
Elbow plot for K‐modes clustering. Total within‐cluster distances between number of clusters was 410, 245, 208, 173, 175, 154, 139, 125, 132, 134. A three‐cluster solution was considered the most appropriate.

To identify the underlying construct and to determine whether the initial, mild, moderate and severe categories can be discriminated from each other based on variables suggested in the classification, cluster analysis was performed. This demonstrated a 3‐cluster solution to be the most informative (Figure 1). The association of the variables within each category used in Wolters classification was then assessed and some but not all variables were shown to significantly discriminate category membership (Table 3). Variables such as sleep disturbance (*p* < 2.2e−16), prolonged response to cold test (*p* < 2.2e−16), nature of pain (*p* = 1.033e−15), severity of response to cold test (*p* = 4.105e−09) and caries depth (*p* = 4.931e−10) significantly contributed to the identification of category membership. Pain quality and severity, however, were not predictive (*p* > .05). The association between each cluster and the diagnostic category of mild, moderate or severe was shown to be statistically significant, *p* < 2.2e−16 (Table 4a). Furthermore, the accuracy of a revised Wolters classification model in predicting diagnostic categories was confirmed by calculation of sensitivity, specificity, PPV and NPV. As shown in Table 4b, the revised Wolters classification model demonstrated 100% sensitivity and specificity for accurate classification of mild and severe pulpitis and had 83% sensitivity and 85.7% specificity when classifying moderate pulpitis, as a result of five moderate cases being classified as severe.

**TABLE 3:** Associations of signs and symptoms to the 3‐cluster groups tested using Kruskal–Wallis Rank‐Sum test for continuous variables and Chi‐squared test for categorical variables.

Variables	Cluster 1 (*N* = 35)	Cluster 2 (*N* = 32)	Cluster 3 (*N* = 25)	*p* Value
Caries depth				**4.931e−10**
Deep	4	29	15	
Extremely deep	31	3	10	
Tenderness to percussion				**3.504e−05**
Sensitive	18	32	18	
Not sensitive	17	0	7	
Sensibility severity				**4.105e−09**
Delayed heightened response	3	0	0	
Heightened	4	29	16	
Strong clear heightened	28	3	9	
Sensibility duration				**<2.2e−16**
Not prolonged	0	32	0	
Prolonged	35	0	25	
Pain nature				**1.033e−15**
Asymptomatic	0	6	1	
Spontaneous	35	0	6	
Stimulated	0	26	18	
Pain quality				.**06099**
Dull	3	6	2	
Lancing	4	2	4	
Missing	1	7	1	
Sharp	1	3	3	
Throbbing	26	14	15	
Pain severity (NRS)				.**01457**
Mild	2	13	0	
Moderate	10	18	9	
Severe	10	7	23	
Sleep disturbance				**<2.2e−16**
No	5	32	25	
Yes	30	0	0	
Linger pain and sleep disturbance				**<2.2e−16**
Yes	30	0	0	
All no	0	32	0	
Linger only	5	0	25	

*Note*: “e” indicates powers of 10. Bold values are significance at *p* < 0.05 as indicated in the statistical analaysis section.

**TABLE 4a:** 3K‐modes clusters alignment with the calcification categories, mild, moderate and severe pulpitis is statistically significant, *p*‐value = <2.2e−16; Chi‐squared test.

Cluster	Initial & mild (*n* = 32)	Moderate (*n* = 30)	Severe (*n* = 30)
1	0 (0%)	5 (17%)	30 (100%)
2	32 (100%)	0 (0%)	0 (0%)
3	0 (0%)	25 (83%)	0 (0%)

**TABLE 4b:** Accuracy of the classification based on the 3‐cluster solution. The clustering model performed perfectly for mild and severe, but suboptimally for moderate cases as 5 were classified as severe.

Classification	Sensitivity	Specificity	PPV (%)	NPV (%)
Mild	100% (95% CI: 89.1–100)	100% (95% CI: 89.1–100)	100	100
Moderate	83% (95% CI: 65–94)	85.7% (95% CI: 69.7–95.1)	83	85.7
Severe	100% (95% CI: 88.4–100)	85.7% (95% CI: 69.7–95)	85.7	100

### Predictive validity assessment

In the absence of a gold standard criterion, the predictive validity of the classification was assessed based on the outcome of treatment provided for each category using the chi‐squared test. Outcome data at 12 months was available for 84% (77/92) of patients for this analysis as 15 did not reattend for follow up. As shown in Table 5, and despite higher failure numbers in the moderate and severe pulpitis group, no significant difference was observed between categories (*p* = .29), indicating that the classification reasonably categorized the patients into appropriate groups. Based on the old classification, all patients with irreversible pulpitis (100%; *n* = 60) would have received root canal treatment. Based on the availability of new treatments for pulpitis that allow the tooth to be saved, the new classification enables triaging of cases classified as moderate to receive partial pulpotomy and severe cases to receive either full pulpotomy or pulpectomy where appropriate. Due to this updated classification, only 5% (3/60) of patients who would have previously undergone root canal treatment received this as their first‐line treatment upon clinical presentation. Based on treatment outcome, compared to the old classification, the new classification, in conjunction with new treatments, resulted in preserving 87% (*n* = 67) of the pulps at 1 year.

**TABLE 5:** Predictive validity of the classification based on 12‐month outcome data.

Cluster/category	Success (*n* = 67)	Failure (*n* = 7)	*p* Value
Mild	28	2	.*26*
Moderate	19	4	
Severe	20	4*	

*Note*: The treatment outcome for each category was assessed using the Chi‐Squared test with no significant difference observed. Bold values are significance at *p* < 0.05 as indicated in the statistical analaysis section

* The failure in the severe group included 3 cases that were shown to be partially necrotic and treated with pulpectomy.

## DISCUSSION

This study tested the reliability and clinical validity of Wolters' proposed diagnostic classification for pulpitis based on signs, symptoms and clinical tests (Wolters et al., 2017). This classification is the first to consider the application of VPT as a treatment modality for inflamed pulps that would previously have been treated with root canal treatment or extraction. The study shows that the classification demonstrated good validity, albeit the result of the face and construct validity was suboptimal. An unsupervised cluster analysis indicated that a 4‐category classification may be overfitting the clinical symptoms currently assessed. Instead, a 3‐cluster (category) solution identified by the cluster analysis was found to map with high sensitivity and specificity to a revised Wolter's classification where the initial and mild pulpitis groups are considered as a single category, with moderate and severe pulpitis categories remaining as currently described. Moreover, the predictive validity of the 3‐category solution supports the combination of initial and mild categories and overall model suitability based on treatment outcomes in this study. Whilst the reliability and reproducibility of the classification were shown to be less than that of the AAE classification, it should be noted that the proposed 3‐category classification perfectly stratifies initial/mild and severe pulpitis, but there is some misclassification within the moderate group, indicating the future potential for further clinical refinement of these criteria. Perhaps more studies should look in detail at this group and determine using validation methodology the key signs and symptoms that could accurately determine group membership. In addition, the use of more objective methods to determine the degree of inflammation, such as biomarkers or radiographic depth of caries, may be useful in this regard.

### Reliability assessment

To assess the reproducibility of the classification, the interrater agreement was calculated. This showed an overall ‘fair agreement’ for Wolters et al. (2017) compared with ‘substantial agreement’ when the raters used the AAE (2009). It was clear from the analysis that the raters achieved substantial reproducibility for mild pulpitis, but the reproducibility decreased for initial and severe pulpitis and reduced further for the moderate pulpitis category. It is unclear why the reproducibility was low for the new classification compared with the AAE classification. However, the new classification has more categories (four compared with two), and descriptions provided for the different categories may require more modification based on the validity outcomes of this study. In addition, training and practice of clinicians on the use of the Wolters classification may ensure increased reproducibility in clinical practice.

### Validity assessment

Although validation of scale‐based classifications is considered important (Ford et al., 2007), this is rarely carried out in endodontics. In this study, face validity, which is the simple form of content validity, was tested as the scale had already been proposed by Wolters et al. (2017). Ideally, content validity should have been performed before developing the classification, using expert consensus and statistical approaches (Streiner et al., 2024). When applied to routine practice in this study, the face validity of the classification was not optimal, and this is further confirmed by the cluster (construct) analysis which showed that the construct being measured was not exactly as proposed in the classification. Furthermore, the analysis revealed that not all the variables suggested in the classification contribute to the definition of clusters, including the character of pain and pain severity. In addition, variables such as asymptomatic pulps and extremely deep caries were not included in the Wolters classification but were reported during the history and examination of the study participants and were demonstrated to be important for determining cluster or category membership.

In the cluster analysis, the number of clusters was determined quantitatively using the Elbow method and qualitatively by considering the size of the cluster, the unique features it contains, and how meaningful the cluster is. Here, the 3‐cluster solution was considered the most appropriate because it relates well to the three categories: mild, moderate, and severe pulpitis. A 4‐cluster solution was also considered, and 2 clusters that fit well with the mild and severe categories were produced, but the moderate pulpitis was split into two small categories that were not clinically meaningful. In addition, with our cluster analysis, the variables failed to generate a separate cluster to match the proposed initial pulpitis category in both the 3‐ and 4‐cluster solutions. This is not surprising because there is no clear distinction in the signs and symptoms between initial and mild pulpitis, and both categories received the same treatment of selective caries removal, indicating a possible similar construct. It is worth mentioning here that IPT was suggested in Wolters classification, but as traditionally indirect pulp capping is much more invasive (Kerkhove et al., 1967), we adopted selective caries removal in this study in line with current practice (ESE et al., 2019).

The 3‐cluster solution correctly identified mild and severe pulpitis based on symptoms provided but classified some of the moderate pulpitis cases as severe. This variability is not unexpected for many reasons, including the subjective nature of the variables tested and that inflammation in the dental pulp is a dynamic pathological process that is difficult to define with specific thresholds (Dummer et al., 1980; Seltzer et al., 1963). It could be speculated that the existence of two states within the moderate pulpitis category highlights the transition from a moderate to a severe form of inflammation. The same was also noted for cases that presented with symptoms indicative of severe pulpitis but with a delayed response to sensibility testing that were shown to be partially necrotic on entry to the pulp chamber. These results confirmed the spectrum and complexity of pulpitis that is difficult to capture through subjective symptoms alone and suggest the need for more objective strategies to determine the inflammatory status of the dental pulp using diagnostic biomarkers (Karrar et al., 2023).

The predictive validity of Wolters classification was assessed based on treatment outcomes at 12 months. This approach is highly relevant in the determination of classification system validity, particularly in the absence of true criteria or a gold standard for the disease (Ford et al., 2007). Indeed, our results showed no statistically significant difference between the groups in the outcome of the treatment received, suggesting the 3‐cluster solution categorized the patients appropriately according to their treatment needs. It is imperative to note that more failures reported in the moderate group align well with the compromised construct validity that is reported for this category.

### Strength and limitations of the study

This study is the first to clinically validate the classification proposed by Wolters et al. (2017). It was conducted in line with the standard for validation of classification scales (Ford et al., 2007) and used appropriate methodologies to assess the validity and reliability of the classification. The findings can inform the development of a much‐needed definitive and validated classification of pulpitis (AAE, 2021; ESE et al., 2019). There are some limitations that need to be considered in the interpretation of the results. First, this classification was based on subjective signs and symptoms that do not accurately determine the degree of pulp inflammation. The use of histology as the gold standard in pulp preservation studies (such as this one) is not practical or feasible, and instead, treatment outcome was used as a proxy reference in line with practice (Deyo et al., 1994; Shimada et al., 2010). However, our outcome data only cover the period up to 12 months. Furthermore, the study was conducted in a single geographic location, and taken together, this highlights the need for long‐term multi‐centre studies to validate our findings.

### Modification to the Wolter's classification

The findings from this study highlight some limitations in the proposed classification that need to be considered for future modification to improve the overall validity and reliability of the classification. These are:

1. The lack of strong features to distinguish initial from mild pulpitis suggests the two represent a single construct to be measured. This is further supported by the fact that the two categories received and responded equally to the same treatment.
2. The addition of caries depth is an important variable in determining category membership, particularly for the mild and severe pulpitis groups. Indeed, histological studies confirmed caries penetration depth reflects the pathological status of the pulp, where, unlike deep caries, teeth with extremely deep caries often show severe inflammation, bacterial infection, and ectopic calcification (Demant et al., 2021, 2022).
3. The nature of pain (dull, throbbing, sharp) is not a useful variable for inclusion in the symptom description.
4. Spontaneous lingering pain that is associated with sleep disturbance characterizes severe pulpitis from other categories.
5. The presence of severe spontaneous pain that is sleep disturbing but with a delayed response to the sensibility test. These teeth were found to have partial necrosis, a presentation that was not considered in the classification.

The suggested modifications to the existing classifications are outlined in Table 6.

**TABLE 6:** Proposed modifications to Wolters classification

Diagnosis category	Symptoms/criteria	Revised symptoms/ criteria	Hypothesis/construct	Revised hypothesis/construct	Suggested treatment	Revised suggested treatment
Initial pulpitis	Heightened (++) but not lengthened response to the cold test. Not sensitive upon percussion. No spontaneous pain.	**Initial/mild combined** Heightened response to cold, warmth and sweet stimuli that can last up to 20 seconds but then subsides Not percussion sensitive Stimulated pain, could be asymptomatic No spontaneous pain Deep caries	Not provided	It would be implied that there is limited local inflammation confined to the crown pulp.	Indirect pulp treatment (IPT)	Selective caries removal
Mild pulpitis	Heightened and lengthened reaction to cold (+++), warmth and sweet stimuli that can last up to 20 seconds but then subsides, possibly percussion sensitive (−/+)	As above	It would be implied that there is limited local inflammation confined to the crown pulp.	As above	As above	Selective caries removal
Moderate pulpitis	Clear symptoms, strong, heightened and prolonged reaction to cold, which can last for minutes, possibly percussion sensitive (−/+) spontaneous, dull pain that can be suppressed with pain medication	Heightened and prolonged reaction to cold, which can last for minutes Possible percussion sensitive Stimulated or spontaneous lingering pain that can be suppressed with analgesics. No sleep disturbance Deep/extremely deep caries	It would be implied that there is extensive Local inflammation confined to the crown pulp.	No change	Partial or complete pulpotomy	No change
Severe pulpitis	Severe spontaneous pain Clear pain reaction to warmth and cold stimuli Often, sharp to dull throbbing pain, patients have trouble sleeping because of the pain (gets worse when lying down). Tooth is very sensitive to touch and percussion	Severe spontaneous pain Clear lingering pain reaction to cold and warms stimuli Sleeping disturbance and lingering pain Possible sensitive to percussion Extremely deep caries mostly	It would be implied that there is extensive local inflammation in the crown pulp that possibly extends into the root canals	It would be implied that there is extensive local inflammation in the crown pulp that possibly extends into the root canals or partial necrosis	Complete pulpotomy or pulpectomy depending on control of bleeding. No time limit was set to achieve hemostasis	Complete pulpotomy or pulpectomy depending on control of bleeding and response to sensibility testing. Delayed but lingering response to thermal test may indicate partial necrosis. No time limit was set to achieve haemostasis

## CONCLUSION

Wolters classification represents an important development in pulpitis classification in the era of pulp preservation therapies. Although the results of this study showed its clinical validity, there are limitations that need consideration in a modification of the proposed classification to improve its reliability and confirm its long‐term clinical validity.

## AUTHOR CONTRIBUTIONS

All the authors have made relevant contributions to the manuscript. All the authors have read and approved the final version of the manuscript.

## CONFLICT OF INTEREST STATEMENT

The authors have stated explicitly that there are no conflicts of interest in connection with this article.

## ETHICS STATEMENT

The study was performed in accordance with the ethical standards of the Declaration of Helsinki. All patients gave written informed consent prior to their inclusion in the study. Ethical approval was obtained from the medical campus Institutional Review Board (University of Khartoum) and the Sudanese National Health Research Ethical Committee.

## Data Availability

The data that support the findings of this study are available from the corresponding author upon reasonable request.

## APPENDIX 1

*Reliability assessment group*: Dr. Phil Tomson; School of Dentistry; Institute of Clinical Sciences; University of Birmingham; Email: p.l.tomson@bham.ac.uk. Dr. David Edwards: School of Dental Sciences, University of Newcastle; Email: david.edwards@newcastle.ac.uk. Dr. Jamie Coulter (Newcastle University): School of Dental Sciences, University of Newcastle; Email: jamie.coulter2@newcastle.ac.uk. Dr. Siobhan Cushley (Queen's University Belfast): School of Medicine Dentistry and Biomedical Sciences, Queen's University Belfast; Email: s.cushley@qub.ac.uk. Dr. Roberto Careddu, Trinity College Dublin; Email: drcareddur@gmail.com.
